# Modulation of van der Waals and classical epitaxy induced by strain at the Si step edges in GeSbTe alloys

**DOI:** 10.1038/s41598-017-01502-z

**Published:** 2017-05-03

**Authors:** Eugenio Zallo, Stefano Cecchi, Jos E. Boschker, Antonio M. Mio, Fabrizio Arciprete, Stefania Privitera, Raffaella Calarco

**Affiliations:** 1 Paul-Drude-Institut für Festkörperelektronik, Hausvogteiplatz 5-7, D-10117 Berlin, Germany; 2 Institute for Microelectronics and Microsystems (IMM), Consiglio Nazionale delle Ricerche (CNR), VIII Strada 5, I-95121 Catania, Italy; 3 Dipartimento di Fisica, Università di Roma “Tor Vergata”, Via della Ricerca Scientifica 1, I-00133 Rome, Italy

## Abstract

The present work displays a route to design strain gradients at the interface between substrate and van der Waals bonded materials. The latter are expected to grow decoupled from the substrates and fully relaxed and thus, by definition, incompatible with conventional strain engineering. By the usage of passivated vicinal surfaces we are able to insert strain at step edges of layered chalcogenides, as demonstrated by the tilt of the epilayer in the growth direction with respect of the substrate orientation. The interplay between classical and van der Waals epitaxy can be modulated with an accurate choice of the substrate miscut. High quality crystalline Ge_x_Sb_2_Te_3+x_ with almost Ge_1_Sb_2_Te_4_ composition and improved degree of ordering of the vacancy layers is thus obtained by epitaxial growth of layers on 3–4° stepped Si substrates. These results highlight that it is possible to build and control strain in van der Waals systems, therefore opening up new prospects for the functionalization of epilayers by directly employing vicinal substrates.

## Introduction

Intentional modification of a crystal structure by application of strain has been used for several decades to tune functional properties of materials^1–3^. The strain imposed by the epitaxial mismatch between substrate and epilayer is a prominent approach in semiconductor manufacturing to obtain performance benefit^4,5^. Most crucially, once the film exceeds the critical thickness the elastic energy stored in it relaxes plastically through dislocations formation. On the contrary, in van der Waals (vdW) epitaxy^6^, due to the weak interaction associated with the vdW bonded atomic species, an epilayer grows from the beginning fully relaxed and the lattice matching condition is lifted. This allows for obtaining sharper interfaces and exploiting the new physical phenomena in two-dimensional (2D) heterostructures^7^. On the other hand, the absence of both defects and strain renders the control of some functional properties not accessible. In such materials it would be desirable to modulate between classical and vdW epitaxy by taking advantage of the interaction of the film with the substrate and at the same time maintaining high crystalline quality.

Here, we report on the method to induce strain at the interface in the vdW epitaxy of chalcogenides lying along the pseudo-binary tie line of GeTe-Sb_2_Te_3_^8^ by the employment of vicinal surfaces. The epitaxial layer is tilted along the growth direction with respect to the substrate, due to the out-of-plane lattice mismatch which introduces strain at the step edges, whereas the in-plane component is weakly bonded to the surface. Almost purely Ge_1_Sb_2_Te_4_ (GST124) of trigonal phase with ordered vacancy layers can be obtained on Si substrates with 3–4° miscut. This allows also the in-plane twin domains to be nearly suppressed preserving the epilayer quality.

## Results

### Highly ordered chalcogenides

**Figure 1:**
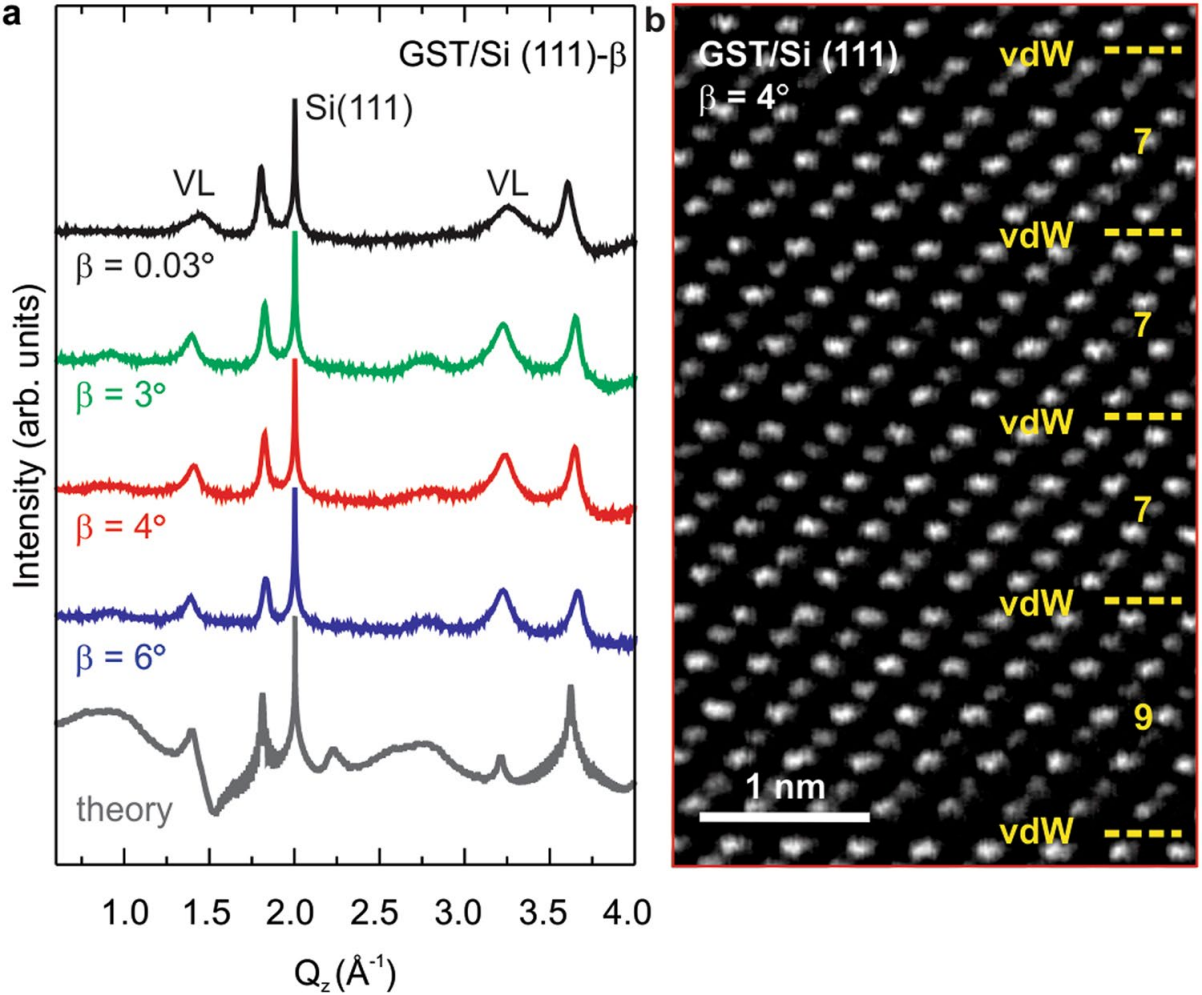
The vicinal surfaces allows for high crystalline quality chalcogenides with reduced structural disorder. (**a**) X-ray diffraction (XRD) ω-2ϑ scans for crystalline GeSbTe (GST) grown on Si (111) with a miscut orientation (*β*) of 0.03° (black), 3° (green), 4° (red), 6° (blue). The substrate miscut improves the FWHM of the vacancy layer (VL) peaks. The gray curve is the simulation of GST grown on miscut. (**b**) Cross-view [11–20] high resolution HAADF-STEM micrograph of epitaxial GST (00.1) on Si (111) with *β* = 4°. Van der Waals (vdW) gaps (dashed yellow lines) occur every 7–9 atomic layers.

The studied GST films were grown by solid source molecular beam epitaxy (MBE) on Si (111) surfaces with a miscut orientation (*β*) of 0.03, 3, 4 and 6° toward the [−211] direction. In order to perform vdW epitaxy, Si surfaces were passivated by Sb to eliminate surface dangling bonds^9^. The detailed sample fabrication is described in Methods. Figure 1a shows the structural investigation by X-ray diffraction (XRD): the symmetric ω-2ϑ scan along the [111] direction for the sample grown on singular Si substrates (*β* = 0.03°, top black curve) reveals the layer to be perfectly (111) oriented with Bragg reflections at Q_z_ = 2.00 Å^−1^ from the Si substrate and two peaks at Q_z_ = 1.80, 3.61 Å^−1^, which are ascribed, respectively, to the (00.15) and (00.30) reflections of GST225^10^. In addition, the two broader features at Q_z_ = 1.45, 3.26 Å^−1^ are interpreted as the first order satellite peaks (reflections (00.12) and (00.27)) of the vacancy layers (VLs); the large width of these two reflections is due to the coexistence of GST blocks with different number of layers^10^. When GST is deposited in the same conditions on vicinal Si surfaces (*β* = 3, 4, 6°, green, red and blue curves) the overall XRD radial profile shows important changes. From the separation between the fully depleted VL peak (vdW gap, see later in the text) and GST epilayer peak average block sizes of 14.95, 14.95, 14.60 Å are obtained for *β* = 3, 4, 6°, respectively, whereas 17.94 Å is the block size for *β* = 0.03°. Also, the degree of ordering, which represents the tendency of some layered materials to feature a single repeating unit in the film^11^, results strongly improved, as shown by the narrower vdW gaps (FWHM from 0.12 to 0.06 Å^−1^ for the VL peak at Q_z_ = 1.45 Å^−1^) and the appearance of second order peaks at Q_z_ = 0.92, 2.77 Å^−1^. The block distribution is even sharper than the case of highly ordered GST realized on singular Si substrates with optimized MBE growth fluxes^10,12^. These findings prove that the substrate miscut reduces the structural disorder in the growth direction with a preferential stacking close to GST124^13^ (*β* = 3–6°), compared to typical GST225^13^ for *β* = 0.03°. Since GST124 is the favorable energetic configuration^13^, it is important to note that the system reaches the equilibrium much faster in presence of the stepped substrate.

Furthermore, a scanning trasmission electron micrograph (STEM) of the sample with *β* = 4° (Fig. 1b) shows that the Te atoms have a rhombohedral stacking sequence^13^ (ABCBC for GST225 and ABCBCABABCAC for GST124) with vdW gaps occurring every 7 (57%, GST124) and 9 (38%, GST225) atomic layers and a very low statistics of 11 layers (GST326). The epitaxial quality is also greatly improved in comparison to annealed amorphous GST samples^12^.

The diffraction profile of GST film on vicinal surfaces has been calculated by using Monte Carlo method^14^ (Methods) with GST ratio 225/124 = 0.6. As shown by bottom gray curve in Fig. 1a, the theory clearly reproduces the main features in the experimental curves for *β* = 3–4°, attesting the validity of the structural model developed here.

### Strain control at the step edges

**Figure 2:**
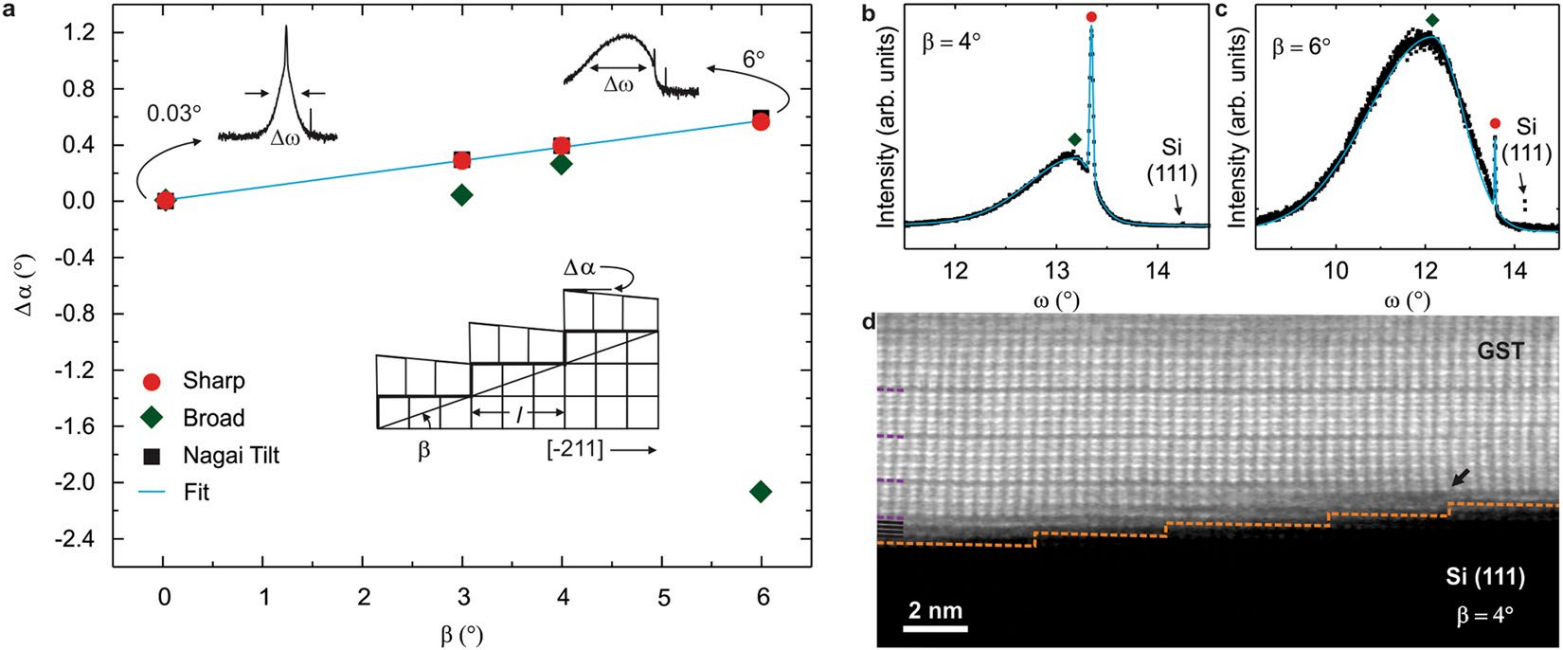
Evidence of strain in vdW bonded materials at the interface between substrate and epilayer. (**a**) Epitaxial orientation (Δ*α*) of the GST obtained from XRD rocking curves as a function of *β*. Two functions are adopted for the fit (red points and green rhombuses for the centers of the sharp and broad peaks, respectively). The Nagai model is represented by the black squares. The inset depicts the modified Nagai model where *l* is the average terrace width. (**b**,**c**) XRD rocking curves centered on GST (00.15) planes at Q_z_ = 1.80 Å^−1^ for *β* = 4 and 6°, respectively. The continuous light blue line is the fit. (**d**) Cross view [−211] high resolution HAADF-STEM micrograph for GST on Si (111) with *β* = 4°. The interface is highlighted by a dotted orange line.

In order to show how the strain can be induced in vdW epitaxy and its role in the growth of GST on vicinal Si surfaces, which conceals the main result of this manuscript, we investigate the out-of-plane orientation of the crystalline film with respect to the substrate. For this purpose, a XRD rocking curve centered at the GST (00.15) peak (Q_z_ = 1.80 Å^−1^) has been measured in two different configurations (Supplementary Fig. 1). When the X-ray beam is parallel to the Si surface steps (along the [−110]) no clear difference is observed in the ω curve: in addition to the weak Si reflection, a GST peak is observed having a sharp component given by the coherently diffracted domains of the single crystalline film and a broader tail originating from the off normal planes. Interestingly, when the X-ray beam is orthogonal to the Si surface steps (along the [−211]) a different scenario emerges. The distribution observed for *β* = 0.03° evolves into an asymmetric one for *β* = 3–6°, where the angular separation between the sharp and broad peaks and the FWHM of the latter increase with offcut angles (insets of Fig. 2a and b). By measuring the separation between the Si peak and the sharp GST peak in the second configuration (X-ray beam along the [−211]), it is possible to quantify the tilt of the film with respect to the substrate. As shown by red points of Fig. 2a, by increasing *β* from 0.03 to 6° the epitaxial orientation (*Δα*) changes away from the surface normal from 0.0003 to 0.6°, respectively. In the case of pseudomorphic growth on off oriented substrates with low defect density at the interface between film and substrate, the epitaxy is ruled by the formula proposed by Nagai^15^, $\tan ({\rm{\Delta }}\alpha )=\frac{{c}_{e}-c}{c}\,\tan (\beta )$, where *c*_e_ and *c* are the lattice parameters of the epilayer and the substrate, respectively, as obtained from XRD data. Strikingly, we find a one to one correspondence of the experimental with the theoretical (black squares, Fig. 2a) data resulting in Δα ≈ 0.094*β* at small *β*. The success of the Nagai description for the tilt of our films is the evidence that strain influences the growth of GST on vicinal surfaces, even though the bonding for the vdW epitaxy is expected to take place by weak interactions at the interface. The out-of-plane mismatch obtained by the Nagai formula, $\frac{\tan ({\rm{\Delta }}\alpha )}{\tan (\beta )}$, increases by a factor of 10 from *β* = 0.03° (1%) to *β* = 3–6° (9.8%), which can be considered an estimation of the strain gradient introduced at the interface by the steps^16^. Such a result is surprising because the Nagai model applies in the case of pseudomorphic growth and a correspondence is not expected in a low defect density vdW system that in its nature is not strongly clamped in the three dimensions. We thus envision a modified Nagai model where the GST is weakly bonded at the terrace, due to the passivation of the Si (111) vicinal surfaces, and grows constrained on both sides of the terrace at the new [−211] orientation defined by the Si step. The main distortion of the lattice is produced in the growth direction and is observable only along the [−211] direction whereas the in-plane matching is expected to be recovered within a terrace (schematic of Fig. 2a). These results show that indeed a strong coupling at kinks and step edges is sufficient to accumulate strain in vdW bonded materials with highly mismatched heterointerfaces allowing for the epilayers to be functionalized from the substrate, while by moving far from the steps the system is mainly vdW like. It is thus possible to modulate the growth of GST from purely vdW epitaxy to a combination of classical and vdW epitaxy by changing *β* from 0.03° to 3–6°, a result never reported before. Important information can be added by looking at the broad peak of the rocking curves (Fig. 2b and c). This has been fitted with an asymmetric pseudovoigt function^17^ and from the obtained values we find that *Δα* also increases up to *β* = 4° (green rhombuses, Fig. 2a). Interestingly, the slope changes sign at *β* = 6° and the tilts reach large negative values, which might be a signature of the largest interaction between film and substrate. In addition, the in-plane lattice constant decreases from 4.22 to 4.04 Å (Supplementary Fig. 2).

To unravel the underlying mechanism for the growth on vicinal Si surface high resolution STEM study was performed on the sample with *β* = 4°, as shown by Fig. 2d. The interface can be well resolved with the stepped substrate highlighted by the orange dotted line: about five terraces are visible in the micrograph (average width *l* ~44.8 Å, height = 3.13 Å) and the intensity contrast is due to the different atomic number of Si and GST. In particular, the brightest atoms are Sb or Te and the gray ones are Ge, and the terrace width ranges from *l* = 50.5 to 37.5 Å (third and fourth terraces from left to right, respectively). By observing the first terrace the GST film sticks on the Si and the lattice planes are well aligned. The stacking shows that VL ordering is obtained very fast with the formation of vdW gaps after few layers (~4, violet dotted lines) and the blocks have mainly GST124 composition. Moreover, the fourth and fifth terraces evidence that the epilayer does not grow properly and two planes are merging in one forming a dislocation at the step position (arrow in Fig. 2d). The epilayer thus shows an opposite tilt with respect to the substrate miscut and the vdW gaps are interrupted due to the change of block distribution, which increases the compositional disorder. These stacking defects form with the appearance in the rocking curve of shifted broad peaks when *β* > 0.03° and become significant at *β* = 6°. The increase of the off normal planes for 30 Å wide terraces at *β* = 6° is evidenced by the more pronounced broad peak with respect to the single crystalline one (Fig. 2c) and might be attributed to the larger number of steps.

The adoption of Si substrates with *β* = 3–4° for the chalcogenide growth thus allows for the introduction of strain at the interface and the epilayer exhibits high overall crystalline quality and mainly GST124 composition, which is able to fast relaxing the strain in the system.

### Suppression of twin domains

**Figure 3:**
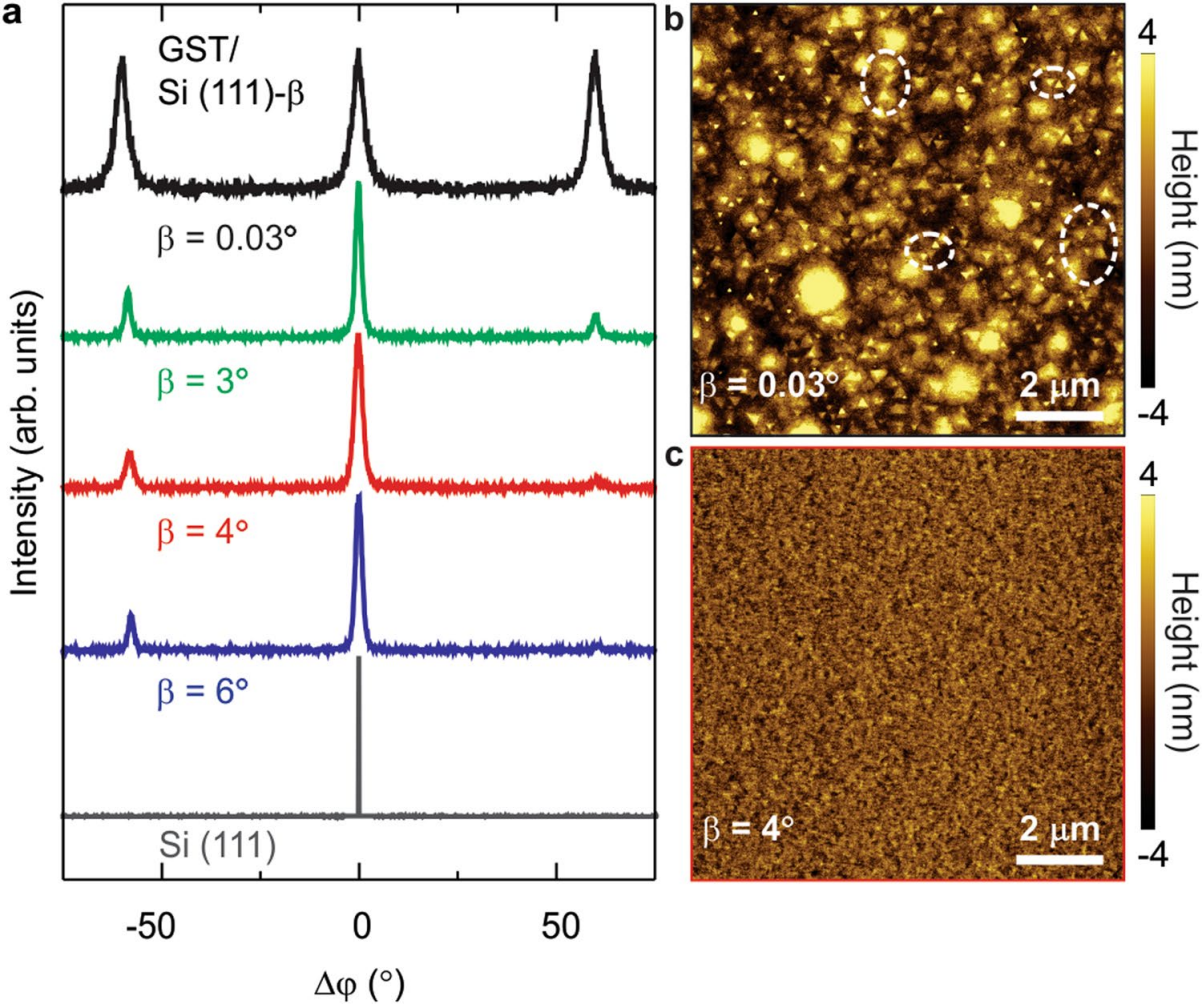
Suppression of GST twin domains by the effect of the Si step edges. (**a**) Φ scans around GST (01.13) grown on Si (111) with *β* = 0.03° (black), 3° (green), 4° (red), 6° (blue). The twinnings are nearly suppressed for *β* = 3–6°. (**b**,**c**) AFM topography (10 × 10 µm) for the samples with *β* = 0.03 and 4°, respectively.

In the following, we show how the growth on vicinal surfaces can suppress the stacking faults - so called twinnings - occurring when there is a shift of atoms stacked on (111) plane from their designated positions. Figure 3a reports the XRD φ-scan of the samples investigated in this work. When GST is grown on singular Si (top black curve) diffraction peaks of the same intensities are measured every 60° whereas the substrate has 3-fold symmetry (bottom gray curve). This is the evidence of GST twin domains formation due to weak interaction at the interface with the substrate^18^, as expected in the case of vdW epitaxy. In fact, by looking at the topography of this sample by atomic force microscopy (AFM), two triangular domains rotated of 180° are visible (white circles, Fig. 3b) and the surface roughness is RMS = 2.3 nm. Contrarily, when the film is realized on the vicinal Si surfaces (curves at *β* = 3–6°, Fig. 3a) the φ = 60° poles are nearly suppressed (~60%), as evidenced in the φ scans reported in Supplementary Fig. 3, due to the growth of one domain over the other boosted by the preferential step flow for the advantageous atomic incorporation at the step edges^19–21^. Strikingly, AFM analysis shows an improved epilayer quality with RMS of only ~1 nm (Fig. 3c), since rougher layers would have been expected for a growth on vicinal surfaces.

### Symmetry of the crystalline phases

**Figure 4:**
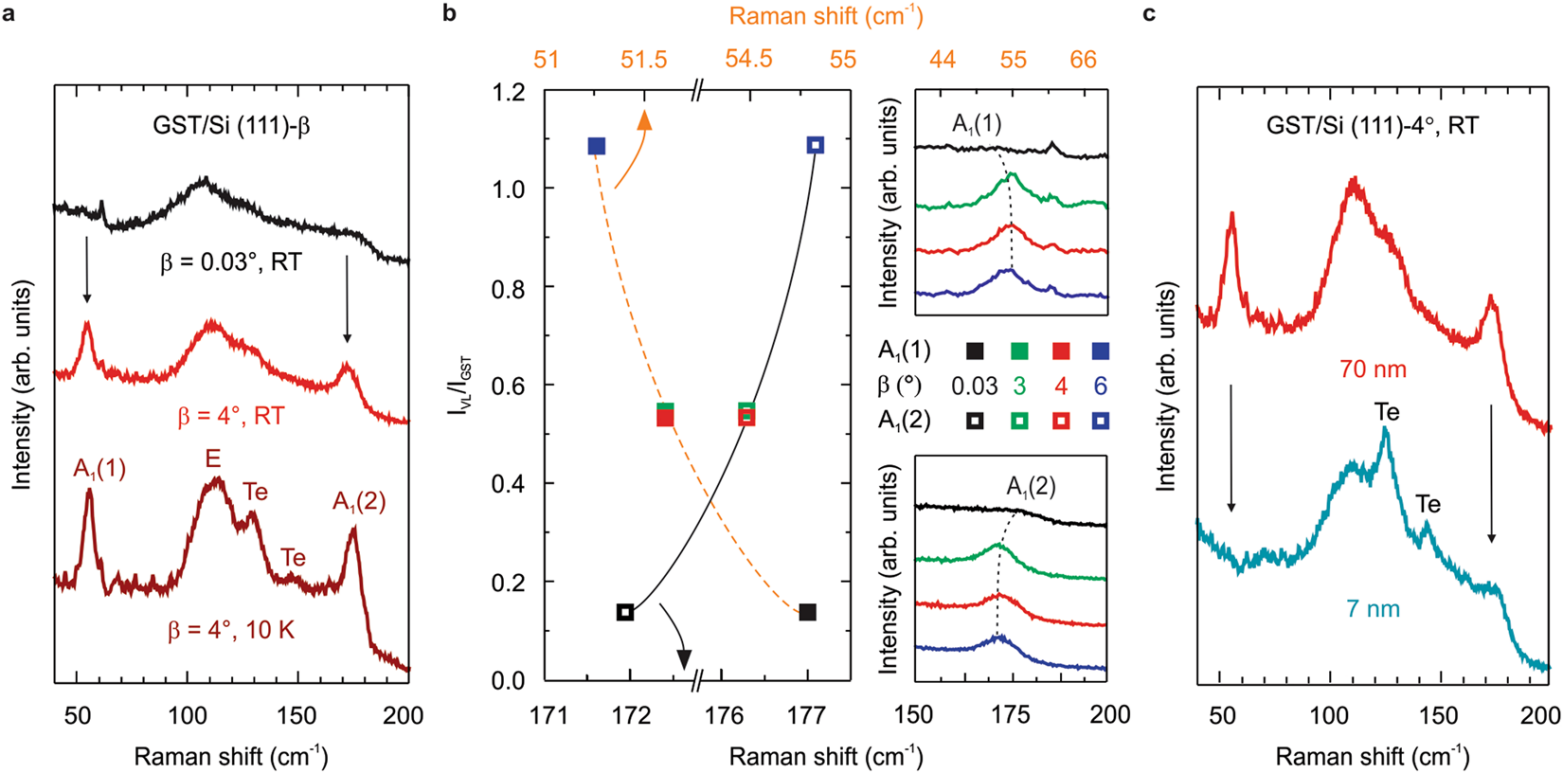
Stable rhombohedral stacking with almost pure GST124 on substrate miscut. (**a**) Raman spectra of 70 nm-thick GST grown on Si (111) with *β* = 0.03° at RT (black), β = 4° at RT (red) and β = 4° at 10 K (dark red). (**b**) Intensity ratio of the second order XRD for the VL peak and GST peak (I_VL_/I_GST_) as a function of the Raman shift for the A_1_(1) (full squares) and A_1_(2) (empty squares) modes with *β* = 0.03° (black), 3° (green), 4° (red) and 6° (blue). Dashed and solid lines serve as a guide to the eye. The top and bottom right panels show the Raman shift of the A_1_(1) and A_1_(2) modes, respectively. (**c**) 70 nm- (red) or 7 nm- (light blue) thick GST grown on Si (111) with *β* = 4°.

In order to get more insight into the crystal symmetry configuration of GST on vicinal Si surfaces and to confirm the high epitaxial quality, a complete Raman study was performed. In Fig. 4a we compare the Stokes scattering for *β* = 0.03 and 4°. The former (black curve) is characterized by a broad mode at ~105 cm^−1^ and a weak bump at ~158 cm^−1^, which correspond to in-plane (E) and out-of-plane (A_1_) vibrations typical of the cubic phase^22^, respectively, and a beginning of VL ordering is represented by the modes at ~53 and ~177 cm^−1^. A closer look to the central E mode evidences a fainter higher frequency component. Remarkably, the *β* = 4° spectrum (red curve) dramatically changes with two narrower modes at 55 and 172 cm^−1^ (A_1_), which are due to the formation of the vdW gaps and are typical of the trigonal GST^23,24^. Similar results are obtained for the other offcut angles thus demonstrating that by introducing strain via stepped Si surfaces it is possible to grow almost pure GST124 with stable rhombohedral stacking. A low temperature (10 K) Raman measurement for *β* = 4° (dark red curve) shows that the full spectrum blueshifts, due to the change in the lattice parameter induced by cooling, and the A_1_(1) mode FWHM decreases from 6.5 to 5.6 cm^−1^. Interestingly, it is now possible to resolve the double peak feature with modes at 129 and 147 cm^−1^, which can be attributed to Te^24^. The total vibrational spectrum resembles the theoretical model of Sosso *et al*.^23^ for ordered GST225 with Kooi stacking sequence^25^. However, the different phase composition (225 vs 124) and the computational details of the model used in ref.^23^ at 0 K can account for the rigid shift of the peaks to higher frequency.

Considering that the formation of the vdW gaps is an important feature of the materials grown by vdW epitaxy, we now report a new way for studying the order in GST alloys by means of a combination of XRD and Raman spectroscopy. The increase in VL ordering with *β* can be clearly visualized by plotting the XRD intensity ratio I_VL_/I_GST_ as function of the Raman shift of the A_1_ modes (Fig. 4b), where I_VL_ and I_GST_ are taken from the VL (00.27) and GST (00.30) peaks, respectively. At *β* = 0.03° the broad VL peak leads to I_VL_/I_GST_ values close to zero, as expected for more disordered GST^10^. However, from *β* = 0.03° to 3–4° the ratio increases to ~0.55 and the FWHM of the vdW gap peaks halves. Interestingly, the A_1_(1) mode blueshifts from 51.4 cm^−1^ to 54.5 cm^−1^ whereas the A_1_(2) mode redshifts from ~177 cm^−1^ to 172.4 cm^−1^. This can be attributed to a change in the layer stacking which affects differently the two out-of-plane vibrations. In addition, I_VL_/I_GST_ > 1 at *β* = 6° since the vdW gaps are still highly ordered but the crystalline GST worsen, as discussed above, and the A_1_(1) and A_1_(2) modes shift respectively to 54.8 and 171.6 cm^−1^. The degree of ordering is thus highlighted also by the shift of the A_1_ peaks, as shown by top and bottom right panels of Fig. 4b.

The VL ordering can be further investigated by comparing the Raman signal of 70 and 7 nm thick samples with *β* = 4° (Fig. 4c). In fact, the spectrum of the latter (bottom light blue curve) is dominated by the peak at ~110 cm^−1^ and the typical out-of-plane vibrational modes of the trigonal phase are strongly suppressed, with the higher frequency one still visible though weak, and a phonon softening is measured due to the decreased film thickness^26^. In addition, Te modes at 125 and 143 cm^−1^ are related to some Te segregation^24^. Since the Raman signal can probe only 16 GST124 (12.5 GST225) blocks^24^, a total number of 5 GST124 (4 GST225) blocks for the thinner sample is probably not sufficient to align perfectly the vdW gaps.

It is thus important to note that the application of appropriate stress on this stable crystal structure realized on substrate miscut together with the suppression of twinning domains might be promising for improving the topological insulating properties in chalcogenides^27,28^.

## Conclusions

In conclusion, we have demonstrated that it is possible to obtain a modulation of vdW epitaxy and classical epitaxy by adopting vicinal surfaces for the growth of vdW bonded materials. The out-of-plane mismatch between the Si substrate and the GST epilayer produces strain accumulation at the step edges resulting in a tilt of the film, which is well reproduced by the Nagai model. By depositing epitaxial GST layers on 3–6° stepped Si substrates, GST with stable trigonal phase, ordered vdW gaps and nearly suppressed twin domains can be obtained. XRD and STEM measurements evidence that the compositional disorder is strongly reduced in presence of miscut and is almost purely GST124. On the other hand, when a similar growth procedure is employed on singular Si surfaces the material grows relaxed from the beginning as typical for the vdW epitaxy. In this way, a specific choice of substrate offcut angles suppresses the compositional dispersion, improves the epilayer quality by controlling the formation of defects in the growth direction and ultimately allows for realization of functionalized films. The vdW epitaxy of chalcogenide-based alloys can be thus finally engineered and the strain induced at the step edges opens up new perspectives for careful growth control.

## Methods

### MBE growth

The studied samples consist of 7 and 70 nm GST films grown by solid source MBE on Sb-passivated surfaces of Si (111), (√3x√3)R30°-Sb, with 0.03, 3, 4, 6° miscut and substrate temperature of 227.5 °C. All the substrates are cleaned and the surfaces are prepared using the methods described in other works^9,29^. According to previous flux calibration by X-ray reflectivity (XRR) measurements on amorphous Ge, Sb, and Te films grown at room temperature, the cell temperatures correspond to a Ge/Sb/Te flux ratio of ~2/2/5. Reflection high-energy electron diffraction (RHEED) technique was used for confirming the formation of the Si (111)-(7 × 7) reconstruction prior the Sb termination of the substrate.

### XRD

*Ex-situ* structural characterization of the films was performed by high resolution XRD. The system consists of a four-circle PANalytical X’Pert Pro diffractometer equipped with a Ge (220) monochromator and Cu Kα_1_ radiation (λ = 1.540598 Å). For ω-2ϑ scans and rocking curve a 1 mm detector slit was used, whereas φ scans were performed without slit and in skew geometry. The cubic unit cell has been adopted for indexing the XRD pattern of the Si substrate whereas the choice of the hexagonal unit cell in the case of the epilayer accounts for the study of the rhombohedral layered GST structure with space group R-3m.

### Monte Carlo simulations

To interpret the measured diffraction data of GST films grown on vicinal surfaces, random stacking of rhombohedral GST124 and GST225 blocks along the growth direction (as determined by STEM analysis) have been generated using Monte Carlo method. The XRD radial scan was calculated as a function of the GST ratio 225/124 for a number N of simulation samples (N = 100) and then averaged, in order to take into account in-plane disorder within the area probed by the X-ray beam. The GST ratio 225/124 for *β* = 3–4° is 0.6 whereas *β* = 6° contains even higher percentage of 124.

### AFM

The topography of the samples was analyzed by using an AFM (Veeco Nanoscope III) operating in tapping mode.

### HAADF-STEM

JEOL ARM200F Cold FEG STEM working at 200 kV was adopted to obtain high resolution micrographs of the sample. The high angle annular dark field (HAADF)-STEM images were obtained with a convergence semiangle of 33 mrad, allowing for a nominal resolution of about 0.68 Å, with an inner detection semiangle of 83 mrad. Scanning was performed using a dwell time per pixel of 40 μs and a beam current of ≈50 pA. The film was observed in the Si [110] zone axis, corresponding to the GST [11–20] zone axis, in order to directly observe the stacking sequence of the atomic planes. The sample has been also investigated in the Si [−211] zone axis for the study of the interface. HAADF-STEM directly relates the micrograph contrast to the atomic number (Z-contrast), allowing a straightforward interpretation of the images in terms of Ge/Sb and Te layers, plane spacing and vdW gap presence. The sample was prepared by standard cross-sectional mechanical polishing followed by Ar^+^ ion milling at LN_2_ temperature, using a Gatan PIPSII system. Ion energy was set from 2.0 keV to 0.1 keV in order to avoid sample amorphization and damaging.

### Micro-Raman spectroscopy

The Stokes scattering of the samples was calculated by means of micro-Raman spectroscopy. The spectra were excited by a continuous wave He-Ne laser 632.8 nm equipped with a LN2-cooled charge-coupled device (CCD) detector in backscattering geometry. The emission was focused by a microscope objective with 0.9 numerical aperture and the same objective was used for the collection of the signal. The spectral resolution achieved is 0.7 cm^−1^ and a notch filter suppressed the stray light. The vibrational modes were assigned by means of polarization resolved spectroscopy.

## Electronic supplementary material

Supplementary Information
