# Author Correction: Modulation of van der Waals and classical epitaxy induced by strain at the Si step edges in GeSbTe alloys

**DOI:** 10.1038/s41598-018-23156-1

**Published:** 2018-03-19

**Authors:** Eugenio Zallo, Stefano Cecchi, Jos E. Boschker, Antonio M. Mio, Fabrizio Arciprete, Stefania Privitera, Raffaella Calarco

**Affiliations:** 1 Paul-Drude-Institut für Festkörperelektronik, Hausvogteiplatz 5-7, D-10117 Berlin, Germany; 2 Institute for Microelectronics and Microsystems (IMM), Consiglio Nazionale delle Ricerche (CNR), VIII Strada 5, I-95121 Catania, Italy; 3 Dipartimento di Fisica, Università di Roma “Tor Vergata”, Via della Ricerca Scientifica 1, I-00133 Rome, Italy

Correction to: *Scientific Reports* 10.1038/s41598-017-01502-z, published online 03 May 2017

This Article contains errors in Figure 4b. The colours of the curves in the left panel were inadvertently switched. The correct Figure 4b appears below as Figure 1.

**Figure 1:**
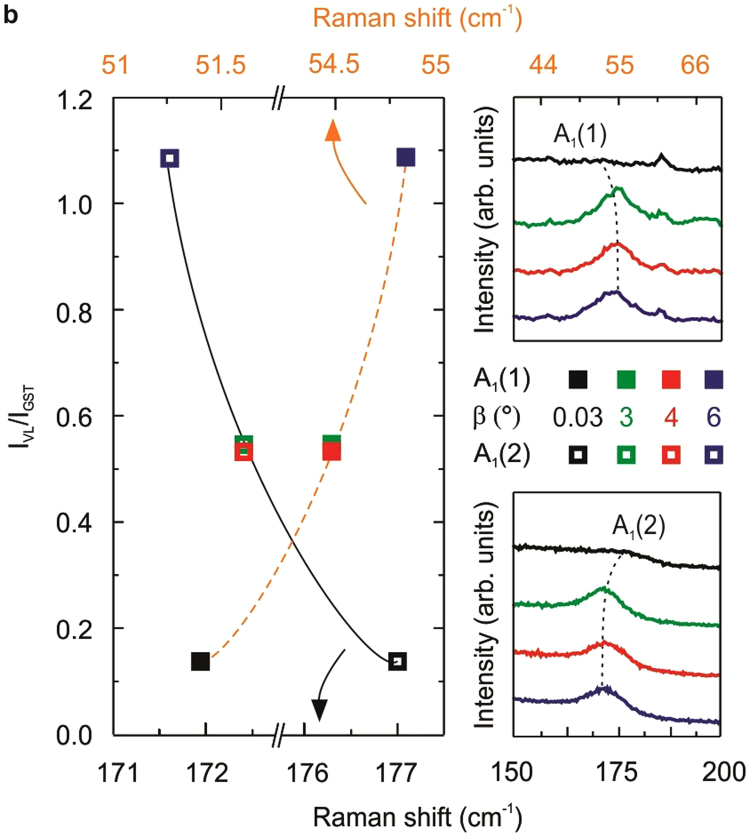
Stable rhombohedral stacking with almost pure GST124 on substrate miscut. (**a**) Raman spectra of 70 nm-thick GST grown on Si (111) with *β* = 0.03° at RT (black), β = 4° at RT (red) and β = 4° at 10 K (dark red). (**b**) Intensity ratio of the second order XRD for the VL peak and GST peak (IVL/IGST) as a function of the Raman shift for the A_1_(1) (full squares) and A_1_(2) (empty squares) modes with β = 0.03° (black), 3° (green), 4° (red) and 6° (blue). Dashed and solid lines serve as a guide to the eye. The top and bottom right panels show the Raman shift of the A_1_(1) and A_1_(2) modes, respectively. (**c**) 70 nm- (red) or 7 nm- (light blue) thick GST grown on Si (111) with *β* = 4°.

