# Supplementary material for: Modulation of van der Waals and classical epitaxy induced by strain at the Si step edges in GeSbTe alloys
Source: Sci Rep. 2017 May 3;7:1466. doi: 10.1038/s41598-017-01502-z (PMC5431103; doi:10.1038/s41598-017-01502-z)
Supplement: Supplementary file 1 — Supplementary Information [file 41598_2017_1502_MOESM1_ESM.pdf]

Supplementary information for:

Modulation of van der Waals and classical epitaxy by strain at the Si step edges in GeSbTe alloys

Eugenio Zallo[1],* Stefano Cecchi[1], Jos E. Boschker[1], Antonio M. Mio[2], Fabrizio Arciprete[1,3], Stefania Privitera[2] and Raffaella Calarco[1]

[1]Paul-Drude-Institut für Festkörperelektronik, Hausvogteiplatz 5-7, D-10117 Berlin, Germany

[2]Institute for Microelectronics and Microsystems (IMM), Consiglio Nazionale delle Ricerche (CNR), VIII Strada 5, I-95121 Catania, Italy

[3]Dipartimento di Fisica, Università di Roma “Tor Vergata”, Via della Ricerca Scientifica 1, I-00133 Rome, Italy

Corresponding Author

Eugenio Zallo

Paul-Drude-Institut für Festkörperelektronik

Hausvogteiplatz 5-7, 10117 Berlin, Germany.

Tel.: +49.30.20377.348 Fax: +49.30.20377.201

e-mail: zallo@pdi-berlin.de

X-ray diffraction (XRD) rocking curves:

XRD rocking curves centered at the GST (00.15) peak have been measured with the X-ray aligned parallel (along the [-110]) and orthogonal (along the [-211]) to the substrate steps. By increasing the miscut from $\beta = 0.03$ to 4° in the parallel configuration (Supplementary Figs. 1a and 1c), the broadening of the sharp peak representing the coherent diffracted domains of the single crystalline film remains constant (~0.07°) whereas the off normal planes peak shows a change of the FWHM from 0.53 to 0.59°. In the orthogonal configuration (Supplementary Figs. 1b and 1d) the symmetric distribution for $\beta = 0.03°$ converts to an asymmetric one for $\beta = 4°$ with the crystalline peak having a narrower FWHM of 0.04° and the broader peak that has been fitted with an asymmetric pseudovoigt function (FWHM = 1.17 and 0.40°).

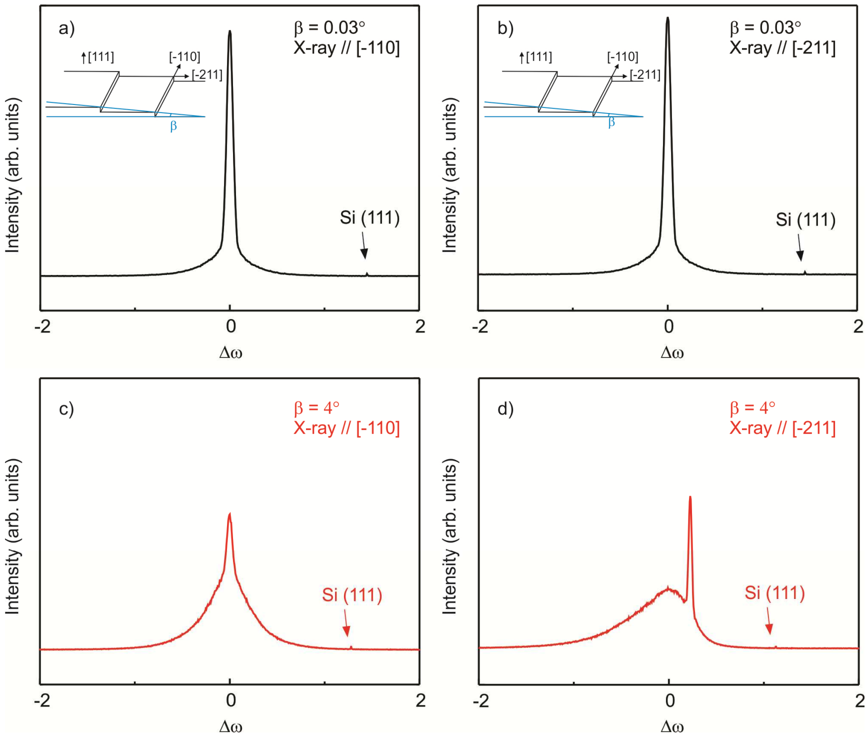

Supplementary Figure 1 XRD rocking curves centered on GST (00.15) planes for β = 0.03° (a, b) and β = 4° (c, d) when the X-ray beam is parallel to the (a, c) [-110] and (b, d) [-211] Si substrate directions (see the schematics).

In-plane lattice constant:

The reciprocal space maps around the asymmetric Si (224) Bragg peak in coplanar configuration are shown in Supplementary Figs. 2(a-c). The Q_z and Q_y axes are parallel to the Si [111] and [-1-12] directions, respectively. From the coordinates of the GST (01.13) peaks with respect of the Si (224) it is possible to extract the in-plane lattice constant ($a_{//}$) of the GST epilayer as a function of the substrate miscut. In particular, from $\beta = 0.03$ to 3° there is no significant change of $a_{//}$ (Supplementary Figs. 2a and 2b). Interestingly, a decrease of $a_{//}$ from 4.22 to 4.04 Å is visible in Fig. 2c for the $\beta = 6°$ case, which is the signature of the large strain introduced in the structure. Moreover, the reduction of compositional disorder associated with the evolution of the film composition towards the 124 phase for $\beta \geq 3°$ is nicely depicted.

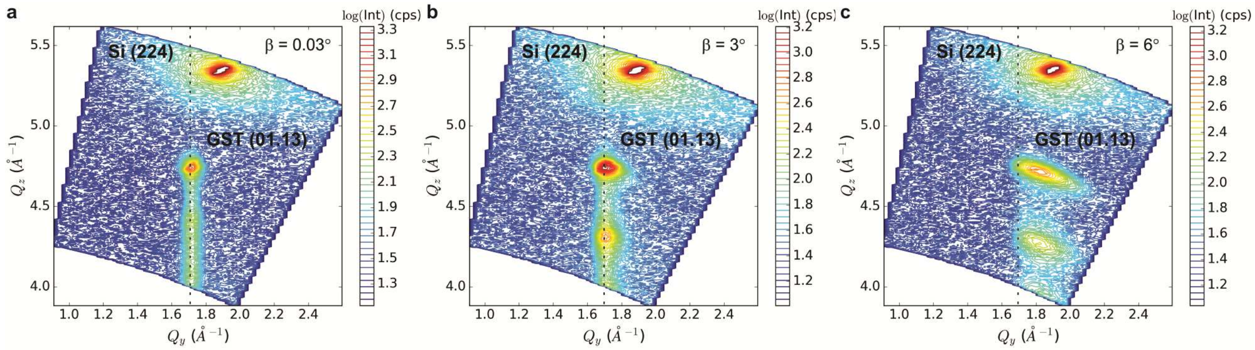

Supplementary Figure 2 Reciprocal space map around the asymmetric Si (224) Bragg peak with Q_z // Si [111] and Q_y // Si [-1-12] for GST grown on Si (111) with $\beta = 0.03°$ (a), 3° (b) and 6° (c).

Φ scan:

The goniometer of the XRD has been rotated (ψ-angle) in order to compensate for the substrate miscut and measure the precise peak intensity. A ψ-φ map is reported in Fig. 3 for β = 4° where the distance along the ψ-axis between each pole is ~4°. As a result, a suppression of the peak intensity at φ = 60° of ~60% is obtained.

It is important to note that the FWHM of the XRD peaks decreases from 3.1° at β = 0.03° to 1.7, 2.2 and 1.9° at β = 3, 4 and 6°, respectively (Fig. 3a of the main text). This is the signature of the better in-plane domain alignment in the case of miscut, which can be related to the higher interaction of the epilayer with the substrate.

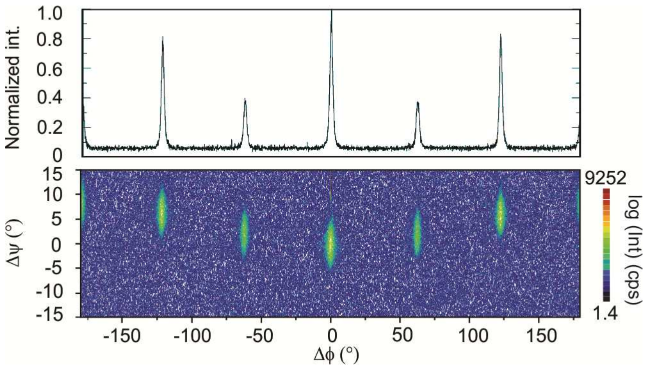

Supplementary Figure 3 XRD ψ-φ map around GST <01.13> for β = 4°. The top panel shows the normalized intensity integrated for different ψ-values.
